# Ophidiomycosis, an emerging fungal disease of snakes: Targeted surveillance on military lands and detection in the western US and Puerto Rico

**DOI:** 10.1371/journal.pone.0240415

**Published:** 2020-10-08

**Authors:** Matthew C. Allender, Michael J. Ravesi, Ellen Haynes, Emilie Ospina, Christopher Petersen, Christopher A. Phillips, Robert Lovich

**Affiliations:** 1 Wildlife Epidemiology Laboratory, Veterinary Diagnostic Lab, Department of Veterinary Clinical Medicine, College of Veterinary Medicine, University of Illinois Urbana-Champaign, Urbana, IL, United States of America; 2 Illinois Natural History Survey, Prairie Research Institute, University of Illinois Urbana-Champaign, Champaign, IL, United States of America; 3 Wildlife Division, Connecticut Department of Energy and Environmental Protection, Sessions Woods WMA, Burlington, CT, United States of America; 4 Naval Facilities Engineering Command Atlantic, Norfolk, VA, United States of America; 5 Naval Facilities Engineering Command Southwest, San Diego, CA, United States of America; Vanderbilt University School of Medicine, UNITED STATES

## Abstract

Wildlife disease surveillance and pathogen detection are fundamental for conservation, population sustainability, and public health. Detection of pathogens in snakes is often overlooked despite their essential roles as both predators and prey within their communities. Ophidiomycosis (formerly referred to as Snake Fungal Disease, SFD), an emergent disease on the North American landscape caused by the fungus *Ophidiomyces ophiodiicola*, poses a threat to snake population health and stability. We tested 657 individual snakes representing 58 species in 31 states from 56 military bases in the continental US and Puerto Rico for *O*. *ophiodiicola*. *Ophidiomyces ophiodiicola* DNA was detected in samples from 113 snakes for a prevalence of 17.2% (95% CI: 14.4–20.3%), representing 25 species from 19 states/territories, including the first reports of the pathogen in snakes in Idaho, Oklahoma, and Puerto Rico. Most animals were ophidiomycosis negative (n = 462), with *Ophidiomyces* detected by qPCR (n = 64), possible ophidiomycosis (n = 82), and apparent ophidiomycosis (n = 49) occurring less frequently. Adults had 2.38 times greater odds than juveniles of being diagnosed with ophidiomycosis. Snakes from Georgia, Massachusetts, Pennsylvania, and Virginia all had greater odds of ophidiomycosis diagnosis, while snakes from Idaho were less likely to be diagnosed with ophidiomycosis. The results of this survey indicate that this pathogen is endemic in the eastern US and identified new sites that could represent emergence or improved detection of endemic sites. The direct mortality of snakes with ophidiomycosis is unknown from this study, but the presence of numerous individuals with clinical disease warrants further investigation and possible conservation action.

## Introduction

Global landscapes have experienced unprecedented changes, largely due to anthropogenic activities, and many habitats no longer resemble the ecosystems in which species evolved [1]. These landscape changes are associated with population and species declines due to several factors, including habitat destruction, climate change, and infectious disease [1–6]. Deteriorating ecosystem health further threatens the sustainability of natural resource management [7], the prevention of human disease [8, 9], and the success of efforts to conserve biodiversity [10, 11].

Snakes fulfill numerous important functions for maintaining healthy ecosystems and subsequently promoting global health. As generalist predators, they control populations of small mammals and therefore aid in controlling the spread of zoonotic diseases including viruses such as hantavirus and bacteria such as Lyme disease [12]. It was observed that Timber Rattlesnakes’ small mammal consumption was responsible for the removal of 2500–4500 ticks per sampled site per year, which was proposed to reduce the incidence of Lyme disease in the areas where these snakes live [13]. Furthermore, healthy snake populations promote overall biodiversity, which is integral for maximizing ecosystem health and productivity [14]. Three hundred and nineteen snake species world-wide, representing 15% of known species with a population assessment, are at risk due to global landscape changes [15].

Natural resources on military lands support a large percentage of endangered habitats and species in the US [16, 17]. Amphibian and reptile species present on Department of Defense (DoD) sites represent 66% of the total native species documented in the continental US [18]. The DoD manages herpetofauna under guidance from a comprehensive strategic plan [19] and implements an overall ecosystem management approach to maintain and/or restore healthy land and water habitats in support of military training [20]. Military lands are home to 131 snake species, several either currently listed or candidates for listing as threatened or endangered by the USFWS (e.g. Eastern Indigo Snake, Louisiana Pinesnake, Black Pinesnake, Brown Gartersnake, Eastern Massasauga) [18]. Therefore, understanding potential threats to these species is of conservation interest and may have implications for military training activities in which federal natural resource management regulations play a role.

Investigating pathogen occurrence in amphibians on DoD lands has aided in conservation missions [21–23], but to date, large-scale efforts have not been made to investigate the health of snakes. Ophidiomycosis (formerly known as Snake Fungal Disease; SFD) is an emerging infectious disease of wild and captive snakes that poses a potential threat to snake biodiversity [24, 25]. Experimental infection studies have determined that the disease is caused by the keratophilic fungus *Ophidiomyces ophiodiicola* [26, 27], and the disease has been observed in more than 30 species of snakes in the US and Europe [24, 28–30]. Interest in monitoring the prevalence of SFD has increased since its implication in the declines of populations of Timber Rattlesnakes (*Crotalus horridus*) [31] and Eastern Massasaugas (*Sistrurus catenatus*) [32]. Clinical signs of SFD include accelerated ecdysis cycles, epidermal flaking and crusting, displaced and/or discolored scales, granulomas, nodules, and swelling or disfiguration of infected tissues [33].

Molecular diagnostics are increasingly important tools for understanding the epidemiology of infectious diseases of wildlife, specifically reptiles [34–36]. The significant variation in the presentation of ophidiomycosis across species and between individual snakes [24, 33] necessitates highly sensitive, non-invasive methods for detection. Fortunately, a combination of qPCR from skin swabs [37] and clinical signs have been shown to be a good predictor of *O*. *ophiodiicola* [38, 39], but this combination assessment may still have a high false negative rate [40]. However, swabbing was found to be in good agreement with scale clips and is less invasive [39]. Recently, a case definition for ophidiomycosis was published that establishes criteria for ophidiomycosis categories, utilizing the results of qPCR from swab samples in combination with clinical signs [33]. The inclusion of clinical signs in a scoring system has been established for Pygmy Rattlesnakes (*Sistrurus miliarius*) [41], but evaluation of its utility across other species is needed.

Our project specifically set out to: 1) Determine the occurrence of ophidiomycosis on DoD lands across the US and its territories using qPCR in combination with clinical signs; and 2) Determine the significant demographic, spatial, or taxonomic associations with ophidiomycosis. These data will provide important health data for snakes on DoD sites. In addition, characterizing the prevalence of ophidiomycosis and its causative agent, a pathogen with conservation and potential public health significance through its impacts on snake population health, will allow assessment of critical spatial and taxonomic variability. This can inform management efforts to minimize the impact of this pathogen on all snake populations, particularly imperiled species.

## Materials and methods

### Field sample collection

*Ophidiomyces ophiodiicola* sampling kits were provided to 68 military installations, and samples were received from 56 for a response rate of 82%. Field surveys of snakes for ophidiomycosis began on 1 April 2018, and samples were collected through 31 December 2018. Scientific names follow Crother [42]. Subspecies were assigned only when it was clear from collecting location. Similarly, sex and age class were assigned when a clear class was known by biologists on the base, otherwise sex and age class were classified as unknown. Upon capture, all snakes were visually inspected for skin lesions, including scabs or other areas that may indicate an *O*. *ophiodiicola* infection [33]. Ophidiomycosis status was assigned based on a recently published case definition [33]. Categories included: 1) Negative (no skin lesions or qPCR detection of *O*. *ophiodiicola* DNA); 2) *Ophidiomyces* present (qPCR detection in absence of clinical signs); 3) Possible ophidiomycosis (presence of clinical signs in absence of qPCR detection); and 4) Apparent ophidiomycosis (presence of clinical signs and qPCR detection).

After visual inspection, swab samples were collected from all individuals using sterile cotton-tipped applicators. Clean handling procedures (i.e., a combination of wearing rubber gloves and sanitizing hands and processing equipment with an alcohol or bleach solution) were used while collecting samples [43]. We sent a link to a training video, a detailed written protocol, and study-specific data sheets to each collaborator to standardize field sample collection. Animal use for the training video was approved by the University of Illinois IACUC (protocol #17046). Each installation was required to obtain the appropriate permit for collection on government lands prior to starting work. For snakes that had no apparent lesions on the skin, two whole-body swab samples were collected by making eight passes along the body with each swab, a modification to previously reported methods [40]. If an individual had skin lesions that could indicate ophidiomycosis, additional swabs directly from each affected area(s) were collected, with a maximum of seven lesion swabs from each individual. All swab samples were placed in separate 2.0 ml Eppendorf tubes and frozen within 2 hours until shipment. Demographic characteristics (species, sex, age class) were recorded for each individual.

### Quantitative PCR

DNA extraction and quantitative PCR amplification (qPCR) were performed on swabs as previously reported [37]. DNA extraction followed the manufacturer’s recommendations with the addition of a one hour incubation at 37°C with 12.5U of lyticase prior to the lysis step. Following DNA extraction, each sample was assessed for DNA quantity (measured in ng/μl) and quality (using the ratio of absorbance at 260 nm to 280 nm) using spectrophotometry (Nanodrop, ThermoFisher Scientific). Quantitative PCR was performed in triplicate on a QuantStudio3 real time thermocycler. Samples were considered positive if all replicates had a lower mean cycle threshold (C_t_) value than the lowest detected standard dilution. Copies per reaction were standardized to the total quantity of DNA in the sample by dividing the mean copies/μl for each sample by the DNA concentration, as determined by spectrophotometry.

### Statistical analysis

Prevalence of each ophidiomycosis category was estimated by calculating the 95% binomial confidence interval in total and by sex, age class, installation, and month [44]. Fisher’s Exact test was used to test associations between ophidiomycosis status and each demographic characteristic. Variables with a significance p-value of <0.1 were included in a series of logistic regression models to evaluate the effects of independent variables (species, sex, age class, installation, month) on the output variable (ophidiomycosis category). Dummy variables in the models were established for each categorical variable: species (*Agkistrodon contortrix*), age class (adult), state (Alabama), and installation (Arnold Air Force Base). Since installation and state overlapped, separate models were run that included each of those variables; both variables were not included in any model set. Next, an information theoretic approach was used to determine which model from the candidate set performed best using the AICcmodavg package [45]. All factors and 2-way interactions were included. Higher order interactions were not pursued due to sample size constraints. Odds ratios (OR) were then calculated for significant predictors using epiDisplay package [46]. Normality of standardized DNA copies was assessed using the Shapiro-Wilk test. Mean and 95% confidence intervals were then calculated and compared between species using a Kruskal Wallis test. Positive and negative predictive values were calculated from 2x2 tables to evaluate the usefulness of using skin lesions for detection of *Ophidiomyces*. Statistical significance was assessed at α = 0.05 and all statistical analyses were conducted using commercial software (R Development Core Team, 2016; SPSS ver. 24, Chicago, IL 60606; MedCalc).

### Phylogenetic analysis

A phylogenetic tree was estimated to examine the relationships between the snake species sampled in this study. Nucleotide sequences for the cytochrome B gene for each snake species were obtained from NCBI. Sequences were downloaded and aligned in Geneious 11.1.4 and a phylogenetic tree was generated using the neighbor-joining distance method in the Geneious Tree Builder (https://www.geneious.com). This tree was used to build the phylogeny-based bipartite network as it is consistent with the most recently published snake phylogeny [47].

### Network analysis

Bipartite networks and network projections were created using Gephi software (version 0.9.2) [48]. In the first network, one set of nodes represented the snake species and the second set of nodes represented the four ophidiomycosis categories, as described above. Nodes were linked if snakes of the given species met the criteria of the given ophidiomycosis category, and the link thickness was weighted based on the proportion of snakes of the given species in the given ophidiomycosis category, similar to previous networks created for human diseases and disease genes [49, 50]. In the second network, species were grouped by region and the network was created with link thickness based on the proportion of snakes in each region meeting the criteria for each ophidiomycosis category. Regions were assigned as follows: Southeast US (Alabama, Florida, Georgia, Kentucky, North Carolina, South Carolina, Tennessee), Midwest US (Indiana, Michigan, Minnesota, Wisconsin), Great Plains US (Colorado, Kansas, New Mexico, Oklahoma, Texas, Wyoming), Northeast US (Massachusetts, Maine, New Hampshire, New York), Mid-Atlantic US (Maryland, New Jersey, Pennsylvania, Virginia), Western US (California, Idaho, Nevada, Utah, Washington), and Caribbean (Puerto Rico). Network projections were created using the MultiMode Projections window in Gephi. In the region-region network projections, region nodes were linked if they shared a disease category, and the weight of the connection was proportional to the number of shared categories. In the disease-disease projections, nodes were linked if they were connected to one or more of the same species, with the weight of the link proportional to the number of shared species.

## Results

### General survey results

Snakes were sampled from 56 military installations from 31 states (S1 Table). Quantitative PCR of 1460 swabs was performed from 657 individual snakes representing 58 species (Table 1). There were 430 adults, 121 juveniles, and 106 snakes of unknown age sampled. Some samples (n = 242) arrived at room temperature, rather than being kept cold during shipment, due to a range of shipping issues. Samples that arrived warm (median: 3.70 ng/μl; 10–90 percentiles: 1.96–7.06 ng/μl) had a lower DNA concentration than samples that arrived cold (mean: 4.19 ng/μl; range: 2.18–11.16 ng/μl) (p<0.0001). There was no difference in purity of DNA between samples that arrived warm or cold (p = 0.597). Among samples that were qPCR positive, standardized fungal copy number was non-significantly higher in samples that arrived cold (mean: 456.19 copies/ng DNA) than warm (mean: 168.08 copies/ng DNA) (p = 0.293). Positive samples (mean: 8.76 ng/μl) had a higher concentration of DNA than negative samples (mean: 5.30 ng/μl) (p = 0.013), but there was no difference in purity (p = 0.694).

**Table 1:** Prevalence of *Ophidiomyces ophiodiicola* detected by qPCR in individual snake species on military installations in 2018.

Species	Negative Individuals	Positive Individuals	Prevalence
*Agkistrodon contortrix*	10	3	23%
*Agkistrodon laticinctus*	1	0	0%
*Agkistrodon piscvorus*	7	1	14%
*Arizona elegans*	3	0	0%
*Carphophis amoenus*	10	5	33%
*Chilabothrus inornatus*	6	1	14%
*Chionactis occipitalis*	3	0	0%
*Coluber constrictor*	54	15	22%
*Coluber flagellum*	11	0	0%
*Coluber flagellum piceus*	1	0	0%
*Coluber schotti*	7	0	0%
*Coluber taeniatus*	2	0	0%
*Crotalus adamanteus*	7	12	63%
*Crotalus atrox*	9	0	0%
*Crotalus cerastes*	3	0	0%
*Crotalus horridus*	3	0	0%
*Crotalus oreganus*	15	0	0%
*Crotalus oreganus helleri*	18	0	0%
*Crotalus ruber*	3	0	0%
*Crotalus viridis*	2	0	0%
*Diadophis punctatus*	13	2	13%
*Drymarchon couperi*	5	14	74%
*Farancia abacura*	2	0	0%
*Heterodon platirhinos*	10	0	0%
*Hypsiglena chlorophaea*	1	0	0%
*Lampropeltis californiae*	6	0	0%
*Lampropeltis calligaster*	5	0	0%
*Lampropeltis calligaster rhombomaculata*	1	0	0%
*Lampropeltis getula*	2	0	0%
*Lampropeltis triangulum*	12	4	25%
*Lampropeltis triangulum gentilis*	6	1	14%
*Lichanura trivirgata*	2	0	0%
*Liodytes rigida*	1	0	0%
*Nerodia erythrogaster*	0	2	100%
*Nerodia fasciata*	1	3	75%
*Nerodia rhombifer*	0	1	100%
*Nerodia sipedon*	14	13	48%
*Opheodrys aestivus*	8	1	11%
*Opheodrys vernalis*	2	0	0%
*Pantherophis alleghaniensis*	25	8	24%
*Pantherophis emoryi*	0	4	100%
*Pantherophis guttatus*	5	0	0%
*Pantherophis obsoletus*	7	1	13%
*Pantherophis ramspotti*	0	3	100%
*Pantherophis spiloides*	16	4	20%
*Phyllorhynchus decurtatus*	4	0	0%
*Pituophis catenifer*	62	3	5%
*Pituophis melanoleucus*	9	0	0%
*Pituophis melanoleucus mutigus*	1	0	0%
*Python bivittatus*	1	0	0%
*Rhinocheilus lecontei*	3	0	0%
*Salvadora grahamiae*	1	0	0%
*Sistrurus catenatus*	39	3	7%
*Sonora semiannulata*	2	0	0%
*Storeria dekayi*	9	0	0%
*Storeria occipitomaculata*	2	0	0%
*Thamnophis elegans*	25	0	0%
*Thamnophis hammondii*	1	0	0%
*Thamnophis saurita*	1	1	50%
*Thamnophis sirtalis*	61	7	10%
*Thamnophis sirtalis fitchi*	1	0	0%
*Thamnophis sirtalis infernalis*	1	0	0%
*Virginia valeriae*	2	0	0%
Total	544	112	17%

### Ophidiomyces detection and ophidiomycosis classification

**Fig 1:**
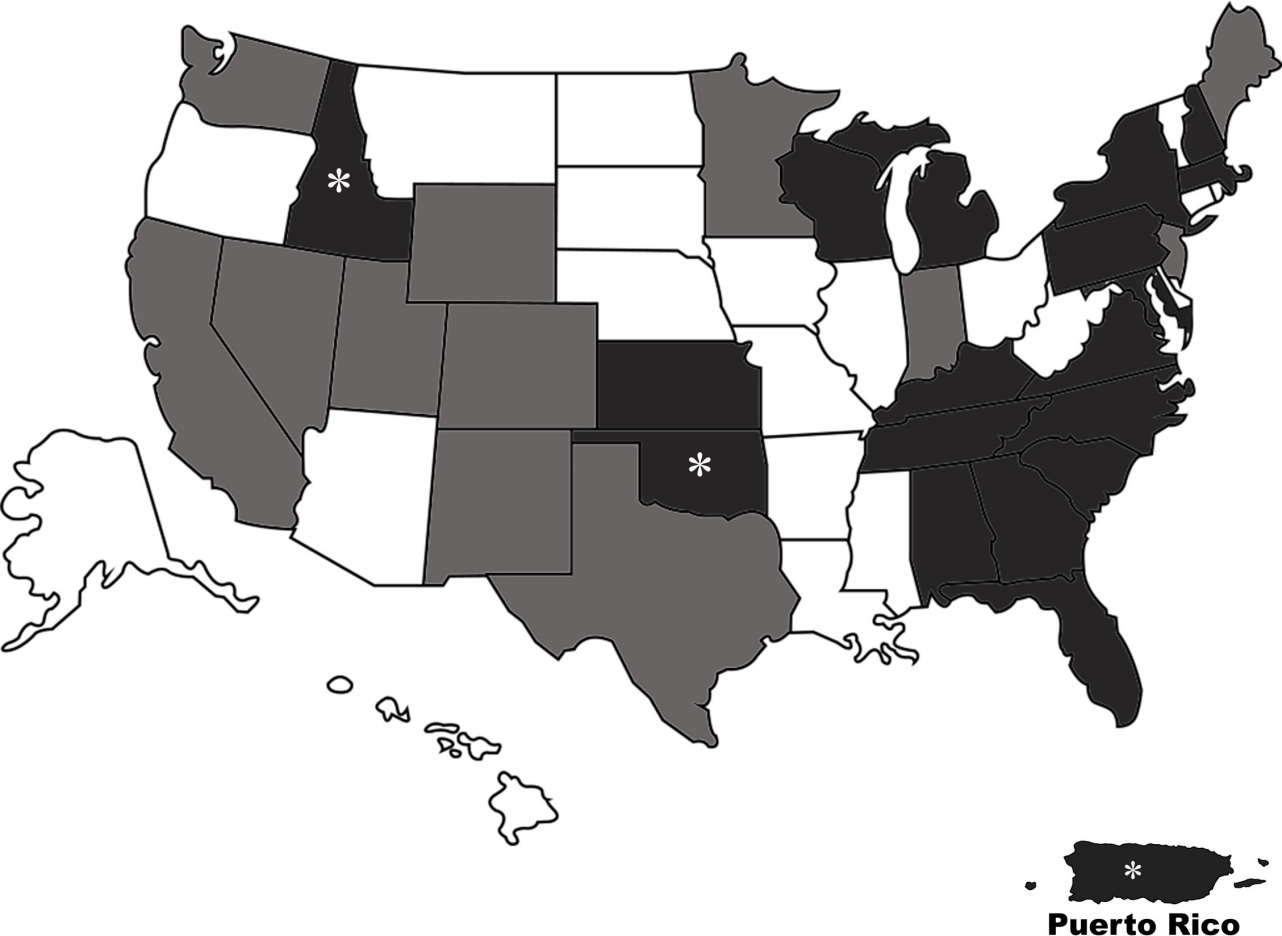
Spatial distribution of *Ophidiomyces ophiodiicola* detection in snakes on military installations sampled in 2018. White = states not sampled, light grey = states with no detection of *O*. *ophiodiicola*, dark grey = states detected with *O*. *ophiodiicola*. White asterisks indicates a state/territory identified with *O*. *ophiodiicola* for the first time in this study.

**Fig 2:**
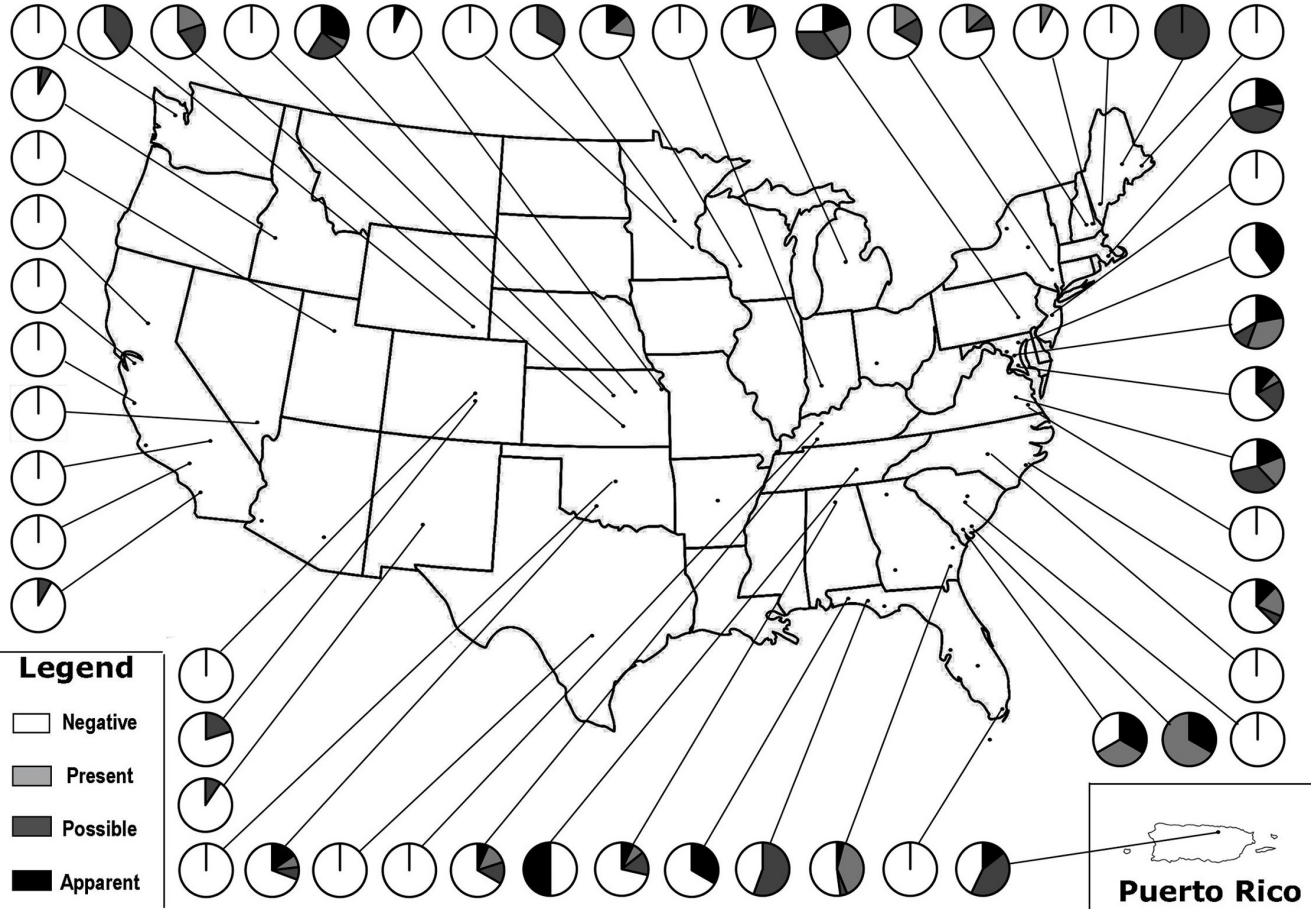
Spatial distribution and prevalence of ophidiomycosis in snakes on military installations sampled in 2018. Negative = no clinical signs and no qPCR detection of *Ophidiomyces ophiodiicola*; (*Ophidiomyces*) Present = qPCR positive AND no clinical signs; Possible (ophidiomycosis) = clinical signs present AND no qPCR detection; Apparent (ophidiomycosis) = qPCR detection AND clinical signs.

Individuals were captured once each and swabbed from 1 to 9 times. Skin lesions were observed in 131 individuals for an overall prevalence of 19.9% (95% CI: 16.9–23.2%). *Ophidiomyces ophiodiicola* DNA was detected in samples from 113 snakes for a prevalence of 17.2% (95% CI: 14.4–20.3%). Nineteen states/territories were detected with *O*. *ophiodiicola* DNA, including for the first time Idaho, Oklahoma, and Puerto Rico (Fig 1). Forty-nine (43.4%) of the qPCR positive individuals had skin lesions. Skin lesions were significantly associated with a qPCR positive result (p<0.0001). The negative predictive value of skin lesions for detecting *O*. *ophiodiicola* DNA was 84.9%, while the positive predictive value was only 43.4%. Most animals were ophidiomycosis negative (n = 462) by qPCR, with *Ophidiomyces* present (n = 64), possible ophidiomycosis (n = 82), and apparent ophidiomycosis (n = 49) occurring less frequently (Fig 2). In states with new detections, Idaho had three snakes with possible ophidiomycosis and one with *Ophidiomyces* present. Oklahoma had two snakes with apparent ophidiomycosis, and one each of possible ophidiomycosis and *Ophidiomyces* present. In Puerto Rico, there was a single individual with apparent ophidiomycosis and three with possible ophidiomycosis. Twenty-three of the sampled species had positive qPCR results and this is the first reported occurrence of *O*. *ophiodiicola* in four of those species: Puerto Rican Boa (*Chilabothrus inornatus*), Great Plains Ratsnake (*Panterhophis emoryi*), Western Milksnake (*Lampropeltis gentilis*), and Western Foxsnake (*Pantherophis ramspotti*).

### Logistic regression models

To increase the predictive power of the models, species with less than 5 individuals represented were removed. Then, a series of multinomial multivariable logistic regression models were fit for ophidiomycosis status (four output categories). The final data set for the model included 595 individuals representing 394 adults, 101 juveniles, and 100 snakes of unknown age. There were 419 negative, 55 *Ophidiomyces* present, 78 possible ophidiomycosis, and 43 apparent ophidiomycosis individuals. The final model explained 96% of the variance (AICc: 637.56, AICc weight: 0.96) with the following variables: state (p<0.0001) and age (p = 0.003). The significant positive predictors of apparent ophidiomycosis included the states Georgia (OR: 5.28, 95% CI: 1.31–21.51, p = 0.027), Massachusetts (OR: 6.0, 95% CI: 1.26–28.55, p = 0.041), and Pennsylvania (OR: 8.75, 95% CI: 1.76–43.6, p = 0.009). Juveniles were 2.38 (95% CI: 1.39–4.17, p = 0.002) times less likely to have ophidiomycosis than adults.

### Network analysis

**Fig 3:**
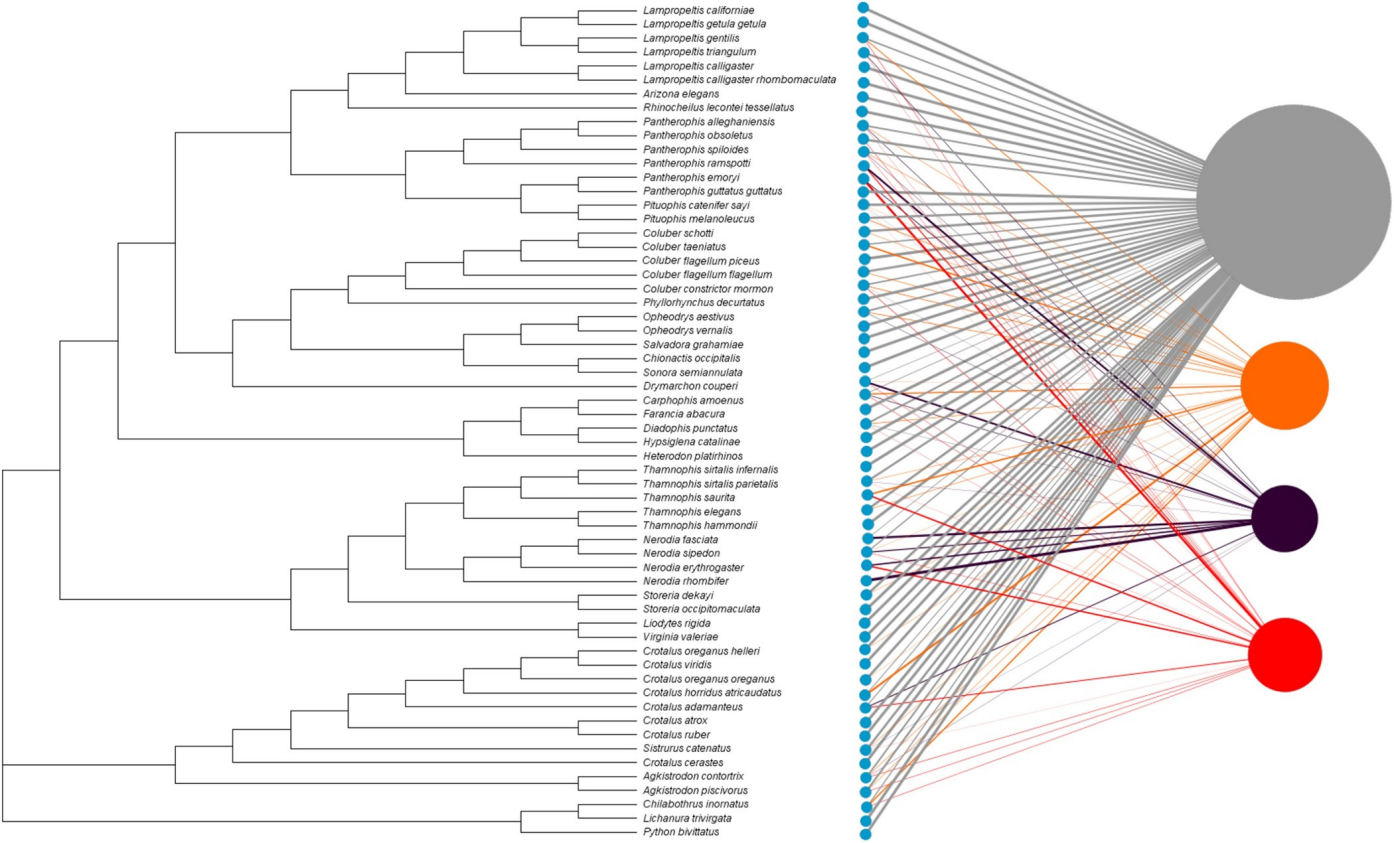
Phylogeny-based bipartite network for snakes sampled for ophidiomycosis on military lands in 2018. Blue nodes represent sampled snake species aligned with the corresponding leaf in the phylogenetic tree. The gray node represents the negative category, the orange node represents possible ophidiomycosis, the dark purple node represents *Ophidiomyces* present, and the red node represents apparent ophidiomycosis. A species node is connected to an ophidiomycosis node if snakes of a given species were classified into the given category. Node size is weighted by prevalence of the ophidiomycosis category, and links are weighted by the proportion of snakes of the given species in the given category.

**Fig 4:**
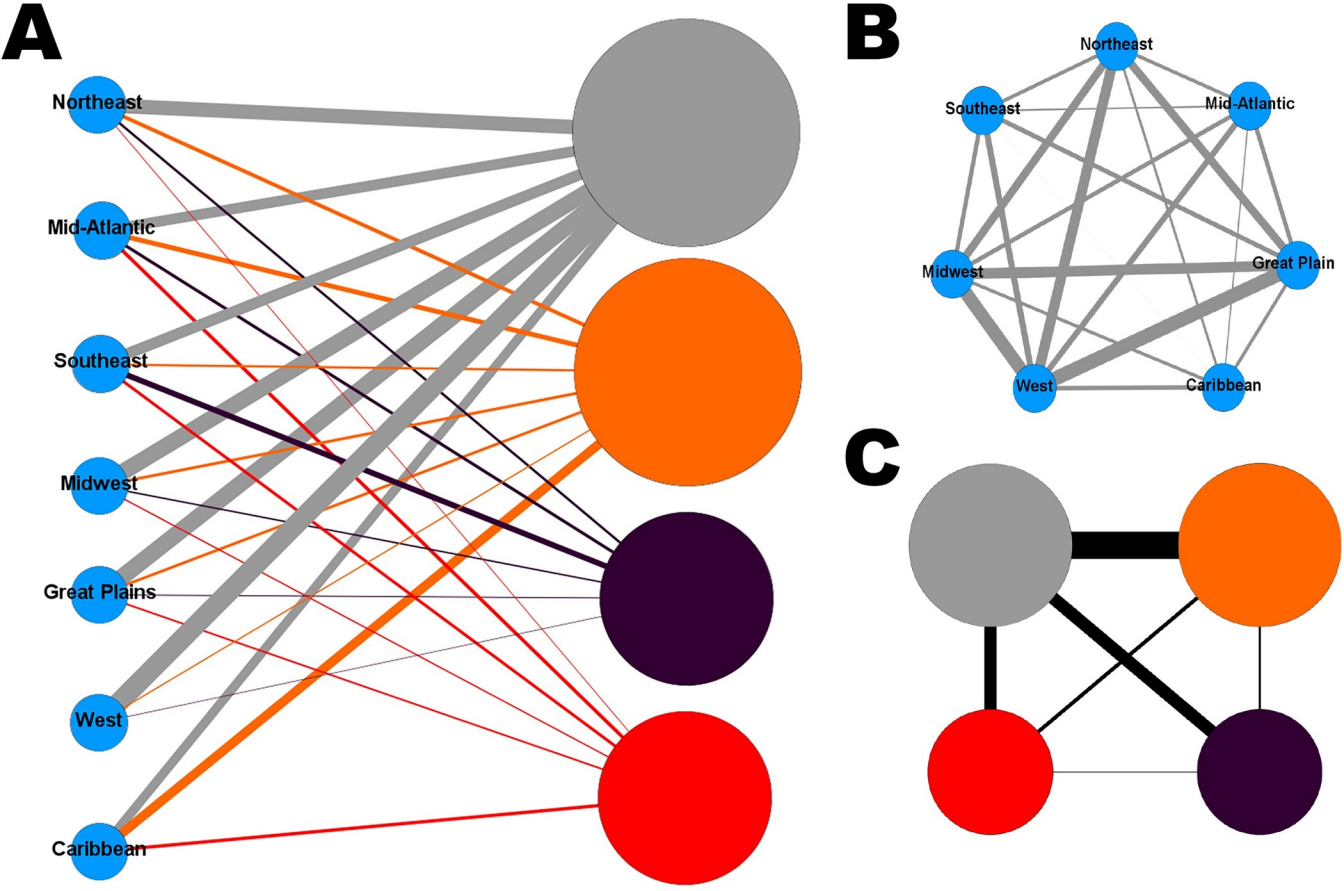
Bipartite network and projections with snake species grouped by region for snakes sampled for ophidiomycosis on military installations. Blue nodes represent regions of the US. The gray node represents the ophidiomycosis negative category, the orange node represents possible ophidiomycosis, the dark purple node represents *Ophidiomyces* present, and the red node represents apparent ophidiomycosis. Ophidiomycosis category node size is proportional to the prevalence of the category. (A) Bipartite network with one set of nodes representing seven US regions and the other set representing ophidiomycosis categories. Nodes are connected if snakes from a given region were classified into the given ophidiomycosis category, and the links are weighted based on the proportion of snakes of the given region in the given category, (B) Region-region network projection with region nodes connected if they share a connection with an ophidiomycosis category, (C) Disease-disease network projection with ophidiomycosis nodes connected if they share a link with a region.

A species network (Fig 3) shows the proportion of snakes in each species that were classified into each ophidiomycosis category, with the species nodes aligned to the phylogenetic tree leaves. A high proportion of all species were classified as negative, thus the largest ophidiomycosis node was the negative category, followed by possible ophidiomycosis, then apparent ophidiomycosis, and finally *Ophidiomyces* present. The strongest connections to the apparent ophidiomycosis group are from *P*. *emoryi*, *P*. *ramspotti*, *T*. *saurita*, and *N*. *sipedon*, while numerous species have no connection to apparent ophidiomycosis category, including the genera *Arizona*, *Rhinocheilus*, *Opheodrys*, *Salvadora*, *Diadophis*, *Liodytes*, and *Storeria*. A bipartite network was then created with two projections based on geographical location (Fig 4A). This network shows connections between every region and every ophidiomycosis category, except that the Caribbean had no snakes in the *Ophidiomyces* present category, and the Western region did not have any connections to apparent ophidiomycosis. Both the region projection (Fig 4B) and the disease-disease projection (Fig 4C) of this network showed high connectivity, but the region-region projection shows the weakest links from the mid-Atlantic region to the southeast US and Caribbean regions. In most cases, snakes of the same species/location fell into multiple ophidiomycosis categories, which resulted in high interconnectedness among nodes in the network projections.

## Discussion

Ophidiomycosis has potentially serious consequences for the success of snake conservation efforts in North America [24, 28], thus threatening biodiversity across several habitats. *Ophidiomyces ophiodiicola* appears to be widespread throughout much of the eastern US and now must be considered a threat to snakes in the western US and Puerto Rico. We detected *O*. *ophiodiicola* DNA in 23 species, four of which (*C*. *inornatus*, *P*. *emoryi*, *L*. *gentilis*, *P*. *ramspotti*) have not been previously reported in the literature. While ophidiomycosis is associated almost exclusively, to date, with skin lesions, infections may also be associated with systemic disease [51, 52]. Skin lesions were not uncommon in this study, and somewhat surprisingly, there were several animals with skin lesions in which *O*. *ophiodiicola* DNA was not detected (possible ophidiomycosis). This may signify the difficulty in sampling for this disease (low DNA quantity on the skin or fungi in tissues deeper than the epidermis). Other confounding factors include the similar appearance of ophidiomycosis lesions to non-infectious skin disease such as trauma, the presence of another pathogen causing similar skin lesions, or a false negative qPCR result due to sample collection or handling.

No species had a significantly higher prevalence of ophidiomycosis than others, but the phylogenetic analysis showed clustering of positive cases among some species of the genera *Nerodia*, *Pantherophis*, and *Thamnophis*. Negative cases were clustered in genera *Liodytes*, *Virginae*, and *Storeria*,. It is not surprising that several species of crotalids were identified with a high prevalence of *O*. *ophiodiicola*, but it is noteworthy that many other crotalid species had no *O*. *ophiodiicola* DNA detected at all. North American crotalids and *Nerodia* species have previously been shown to have a high prevalence of ophidiomycosis and may be uniquely sensitive to infection due to their environment (*Nerodia*) or morphology (pits in crotalids) [24, 28, 39, 53]. Recent studies in aquatic snakes in Kentucky have highlighted that aquatic snake susceptibility is under-represented in the literature and warrants careful investigation [39]. It is possible that the perceived increase in susceptibility of crotalids represents a sampling bias in which more crotalids are sampled historically. The lower observed prevalence in *Liodytes* and *Storeria* may be due to inherent resistance shared among closely related species, the smaller size of the snake resulting in smaller surface area for sampling and subsequent lower DNA quantity, or sharing life history traits or habitats that are less permissive to *O*. *ophiodiicola* infection. Alternatively, the differences in prevalence may actually represent differences in susceptibility associated with habitat characteristics rather than taxonomic class. Future investigations should characterize the mechanisms that lead to lower prevalence of ophidiomycosis in these species or habitats to improve conservation efforts in other sensitive species.

The distribution of ophidiomycosis has been known to extend across the eastern US [24, 28], but we identified cases more recently in the central and western US. It is unclear whether the pathogen is spreading to new areas or being detected in previously untested sites. What is certain is that identifying the reservoirs and modes of environmental transmission is integral to identifying intervention strategies that limit the impacts of the disease at both the individual and population levels.

Age class seems to play a significant role in the pathogenesis of ophidiomycosis as adults were more likely to be detected with *O*. *ophiodiicola* and diagnosed with apparent ophidiomycosis than juveniles. This may be due to the fact that the pathogen is widespread in the environment [28, 39], and adults have an increased exposure to the pathogen over time. Conversely, it is possible that differences in shedding frequency between quickly growing juvenile snakes and adults may reduce exposure time in juveniles, but future studies are needed to determine the causes of lower juvenile prevalence.

The impact of this pathogen on the health of many snake species remains unknown and the true impact on fitness needs to be evaluated. Recently, several investigations have been conducted determining the physiological impact of ophidiomycosis on pygmy rattlesnakes (*Sistrurus miliarius*) in Florida. Specifically, snakes with ophidiomycosis have shown a decrease in body condition with increasing severity of infection, but severity was not associated with recapture rate, and severely affected snakes apparently cleared infection [54]. It was also observed that the resting metabolic rate in this species was increased in ophidiomycosis-positive individuals, which may contribute to declining individual health during the disease process [55]. An increase in fetal mortality was observed following supplemental feeding of ophidiomycosis-positive gravid females [56], and a decrease in sex hormones in both positive males and females was observed, indicating subclinical reproductive effects from ophidiomycosis [57]. Eastern Massasaugas with ophidiomycosis in Michigan were observed moving long distances less frequently and seeking basking sites more often late in the active season, indicating an energetic cost to infection compared to uninfected snakes in the same habitat [58]. If similar effects are observed in other species, the spread of *O*. *ophiodiicola* to vulnerable populations will have significant conservation implications.

The results of this investigation provide important snake health information to participating DoD sites nationwide. Ophidiomycosis epidemiologic investigations have required a collaborative effort between biologists, veterinarians, and land managers, and this study produced a great deal of data about the distribution of this disease. Furthermore, this study provides critical large-scale insight on spatial and taxonomic variability needed to understand how best to minimize disease impact, particularly for imperiled species [8, 9, 11]. However, it is not the only conservation threat to snakes, and may not even be the only disease facing species of conservation concern [24, 34–36]. At a time when wildlife diseases are increasingly important for wildlife populations and public health, and wildlife serve as reservoirs for a wide variety of diseases, the need for early detection, or, ideally, prevention of the next disease event, has never been greater. This study represents an impetus for military installations and other entities to justify wildlife management funding requests, proactively plan and prepare, and take mitigation action, where appropriate. Future health assessments, pathogen detection, and assessment of contaminant exposure in these snake populations may allow us to identify trends and new threats to both snakes and other wildlife species.

## Supporting information

S1 Table Sample sizes and result of *Ophidiomyces ophiodiicola* qPCR testing on military installations, by state, where snakes were sampled in 2018. (DOCX)

S1 Data (XLSX)
