# Supplementary material for: Ophidiomycosis, an emerging fungal disease of snakes: Targeted surveillance on military lands and detection in the western US and Puerto Rico
Source: PLoS One. 2020 Oct 8;15(10):e0240415. doi: 10.1371/journal.pone.0240415 (PMC7544097; doi:10.1371/journal.pone.0240415)
Supplement: S1 Table — (DOCX) [file pone.0240415.s001.docx]

**S1 Table**. Sample sizes and result of *Ophidiomyces ophiodiicola* qPCR testing on military installations, by state, where snakes were sampled in 2018.

| **State** | **Installation** | **Sample size** | **Detected with *O. ophiodiicola*** |
| --- | --- | --- | --- |
| *Alabama* | |  |  |
|  | US Army Garrison Redstone | 14 | 2 |
| *California* | |  |  |
|  | Marine Corps Base Camp Pendleton | 24 | 0 |
|  | Naval Air Weapons Station China Lake | 14 | 0 |
|  | Fort Hunter Liggett | 5 | 0 |
|  | Parks Reserve Forces Training Area | 5 | 0 |
|  | Beale AFB | 4 | 0 |
|  | Edwards AFB | 2 | 0 |
| *Colorado* | |  |  |
|  | USAF Academy | 11 | 0 |
|  | Fort Carson/Pinon Canyon | 10 | 0 |
| *Florida* | |  |  |
|  | Eglin Installation Complex | 9 | 0 |
|  | Homestead AFB | 4 | 0 |
|  | Hurlburt Field | 3 | 1 |
| *Georgia* | |  |  |
|  | Fort Stewart | 35 | 21 |
| *Idaho* | |  |  |
|  | Orchard Combat Training Center | 48 | 1 |
| *Indiana* | |  |  |
|  | Naval Support Activity Crane | 6 | 0 |
| *Kansas* | |  |  |
|  | Fort Riley | 17 | 6 |
|  | Fort Leavenworth | 15 | 1 |
|  | McConnell AFB | 5 | 1 |
|  | Smoky Hill ANG Range | 4 | 0 |
| *Kentucky* | |  |  |
|  | Fort Campbell | 15 | 3 |
|  | Wendell H. Ford Regional Training Center | 3 | 0 |
| *Massachusetts* | |  |  |
|  | Camp Edwards | 17 | 5 |
| *Maryland* | |  |  |
|  | Naval Air Station Patuxent | 35 | 6 |
|  | Blossom Point Research Facility | 9 | 5 |
|  | Navy Support Facility Indian Head | 6 | 1 |
|  | Adelphi Laboratory Center | 5 | 2 |
| *Maine* | |  |  |
|  | Naval Survival, Evasion, Resistance and Escape School | 5 | 0 |
|  | Naval Support Activity Cutler | 2 | 0 |
|  | Great Pond Outdoor Adventure Center | 1 | 0 |
| *Michigan* | |  |  |
| Camp Grayling | | 36 |  |
| *Minnesota* | |  |  |
|  | Camp Ripley | 12 | 0 |
|  | Arden Hills Army Training Site (AHATS) | 3 | 0 |
| *North Carolina* | |  |  |
|  | Marine Corps Base Camp Lejeune | 16 | 5 |
|  | Fort Bragg | 7 | 0 |
| *New Hampshire* | |  |  |
|  | New Boston AFS | 22 | 3 |
|  | Pembroke | 13 | 1 |
| *New Jersey* | |  |  |
|  | Joint Base Maguire-Dix-Lakehurst | 4 | 0 |
| *New Mexico* | |  |  |
|  | White Sands Missile Range | 21 | 0 |
| *Nevada* | |  |  |
|  | Nellis AFB | 17 | 0 |
| *New York* | |  |  |
|  | West Point Military Reservation | 18 | 3 |
| *Oklahoma* | |  |  |
|  | Fort Sill | 13 | 3 |
|  | Tinker AFB | 7 | 0 |
| *Pennsylvania* | |  |  |
|  | Fort Indiantown Gap | 20 | 8 |
| *Puerto Rico* | |  |  |
|  | Fort Buchanan | 7 | 1 |
| *South Carolina* | |  |  |
|  | Parris Island | 21 | 14 |
|  | Fort Jackson Army Installation | 5 | 0 |
|  | Marine Corps Air Station Beaufort | 3 | 3 |
| *Tennessee* | |  |  |
|  | Arnold Air Force Base | 2 | 1 |
| *Texas* | |  |  |
|  | Joint Base San Antonio | 4 | 0 |
| *Utah* | |  |  |
|  | Camp Williams | 5 | 0 |
| *Virginia* | |  |  |
|  | Fort Lee | 21 | 8 |
|  | Fort Eustis | 6 | 0 |
| *Washington* | |  |  |
|  | Joint Base Lewis McChord Yakima | 9 | 0 |
| *Wisconsin* | |  |  |
|  | Fort McCoy | 22 | 6 |
| *Wyoming* | |  |  |
|  | Francis E. Warren AFB | 10 | 0 |
